# Probiotic and Oxytocin Combination Therapy in Patients with Autism Spectrum Disorder: A Randomized, Double-Blinded, Placebo-Controlled Pilot Trial

**DOI:** 10.3390/nu13051552

**Published:** 2021-05-05

**Authors:** Xue-Jun Kong, Jun Liu, Kevin Liu, Madelyn Koh, Hannah Sherman, Siyu Liu, Ruiyi Tian, Piyawat Sukijthamapan, Jiuju Wang, Michelle Fong, Lei Xu, Cullen Clairmont, Min-Seo Jeong, Alice Li, Maria Lopes, Veronica Hagan, Tess Dutton, Suk-Tak (Phoebe) Chan, Hang Lee, Amy Kendall, Kenneth Kwong, Yiqing Song

**Affiliations:** 1 Athinoula A. Martinos Center, Massachusetts General Hospital, Charlestown, MA 02129, USA; Jun_Liu@hms.harvard.edu (J.L.); kliu16@mgh.harvard.edu (K.L.); mkoh@mgh.harvard.edu (M.K.); htsherman@mgh.harvard.edu (H.S.); SLIU41@mgh.harvard.edu (S.L.); RTIAN2@mgh.harvard.edu (R.T.); Jwang106@mgh.harvard.edu (J.W.); Michelle_Fong@DFCI.HARVARD.EDU (M.F.); CCLAIRMONT1@mgh.harvard.edu (C.C.); msjeong@mgh.harvard.edu (M.-S.J.); CLI36@mgh.harvard.edu (A.L.); MLOPES6@mgh.harvard.edu (M.L.); VHAGAN@mgh.harvard.edu (V.H.); tdutton1@bwh.harvard.edu (T.D.); stchan@mgh.harvard.edu (S.-T.C.); alkendall@mgh.harvard.edu (A.K.); KKWONG@mgh.harvard.edu (K.K.); 2 Department of Psychiatry, Beth Israel Deaconess Medical Center, Boston, MA 02215, USA; 3 Harvard Medical School, Boston, MA 02115, USA; Piyawat_Sukijthamapan@DFCI.harvard.edu (P.S.); lei@steele.mgh.harvard.edu (L.X.); hlee5@mgh.harvard.edu (H.L.); 4 Department of Radiation Oncology, Massachusetts General Hospital, Boston, MA 02114, USA; 5 MGH Biostatistics Center, Massachusetts General Hospital, Boston, MA 02114, USA; 6 Department of Epidemiology, Indiana University, Richard M. Fairbanks School of Public Health, Indianapolis, IN 46202, USA; yiqsong@iu.edu

**Keywords:** autism spectrum disorder (ASD), probiotics, oxytocin, microbiome, inflammation markers

## Abstract

Autism spectrum disorder (ASD) is a rapidly growing neurodevelopmental disorder. Both probiotics and oxytocin were reported to have therapeutic potential; however, the combination therapy has not yet been studied. We conducted a randomized, double-blinded, placebo-controlled, 2-stage pilot trial in 35 individuals with ASD aged 3–20 years (median = 10.30 years). Subjects were randomly assigned to receive daily *Lactobacillus plantarum* PS128 probiotic (6 × 10^10^ CFUs) or a placebo for 28 weeks; starting on week 16, both groups received oxytocin. The primary outcomes measure socio-behavioral severity using the Social Responsiveness Scale (SRS) and Aberrant Behavior Checklist (ABC). The secondary outcomes include measures of the Clinical Global Impression (CGI) scale, fecal microbiome, blood serum inflammatory markers, and oxytocin. All outcomes were compared between the two groups at baseline, 16 weeks, and 28 weeks into treatment. We observed improvements in ABC and SRS scores and significant improvements in CGI-improvement between those receiving probiotics and oxytocin combination therapy compared to those receiving placebo (*p* < 0.05). A significant number of favorable gut microbiome network hubs were also identified after combination therapy (*p* < 0.05). The favorable social cognition response of the combination regimen is highly correlated with the abundance of the Eubacterium hallii group. Our findings suggest synergic effects between probiotics PS128 and oxytocin in ASD patients, although further investigation is warranted.

## 1. Introduction

Autism spectrum disorder (ASD) is a complex neurodevelopmental disorder featuring impaired social communication and stereotypical repetitive behavioral patterns. ASD has become a serious health issue due to its rapidly rising prevalence. According to a recent report from the CDC, the prevalence of ASD has risen to 1 in 54 children [1]. However, its etiology remains elusive, and effective treatment is still largely unavailable.

Gut microbiome composition and inflammation have been reported to be involved in the pathogenesis of ASD through the gut–brain axis [2]. Recent evidence demonstrates that alterations in the gut microbiota of ASD individuals changes both gastrointestinal (GI) physiology and behaviors via the gut–microbiome–brain axis [3,4]. Probiotic varieties used in both animal studies and clinical trials have demonstrated efficacy in improving ASD core symptoms [5,6]. Evidence that probiotics have the potential to improve neuropsychiatric symptoms via the gut–brain axis is not limited to ASD. In fact, there is an entire subgroup of probiotics, known as psychobiotics, that may provide health benefits in patients with psychiatric illness [7]. Animal studies have shown some psychobiotic strains can improve depression-like behavior [8], anxiety-like behavior [9], cognition [10], and autism-like behaviors, such as communication defect and stereotypic behaviors [11]. One psychobiotic, *Lactobacillus plantarum* PS128 (PS128), showed ameliorative effects on depression- and anxiety-like behaviors in different mouse models [12,13]. When administered to children with ASD, PS128 was shown to improve anxiety, rule-breaking behaviors, and hyperactivity/impulsivity [5].

Oxytocin (OXT), a neuropeptide produced by the hypothalamus, is well known for its ability to modulate emotional and social communication, bonding, and reward-related behaviors [14]. OXT signaling is implicated in the etiology of ASD, as previous studies using OXT receptor knockout mouse models exhibit autistic-like behavior, such as deficits in social interaction [15]. Subsequent studies have shown that OXT treatment enhanced sociability in two mouse models of ASD [16]. OXT shows promising therapeutic potential for ASD core symptoms because it can be easily administered and can work as a cost-effective treatment with minimal adverse effects. Furthermore, OXT plays an important role in the gut–brain axis and is likely inducible by certain probiotics such as *Lactobacillus reuteri* [14]. However, potential biological connections between *Lactobacillus plantarum*, including PS128, and endogenous OXT have not been studied. Moreover, the interactions between these two promising interventions, OXT, and PS128 have not been tested.

We designed this double-blinded, randomized, placebo-controlled, two-stage pilot trial to test our hypothesis that combination therapy with probiotics and OXT results in a therapeutic synergy that exerts beneficial effects on ASD symptoms. We simultaneously measured the clinical index for ASD core symptoms, gut microbiome profile, and levels of OXT and inflammatory markers in the blood to evaluate the efficacy of the combination therapy and identify impacting factors with predictive value for treatment outcomes.

## 2. Materials and Methods

### 2.1. Trial Design

This clinical trial is a randomized, double-blind, and placebo-controlled study in accordance with the Consolidated Standards of Reporting Trials (CONSORT) guidelines. Subjects were randomized to two groups with a 1:1 ratio into this two-stage study. To achieve a statistical power of 80% for primary outcomes with a large effect size of 0.8 (Cohen’s d) assumed, a total of 60 participants (30 in each arm) were required. However, as we are primarily interested in studying the preliminary effects of the proposed treatment, we enrolled and randomized 35 subjects who were included in the data analysis. In stage 1, the probiotics group received oral probiotics PS128 while the placebo group received oral placebo for 16 weeks. In stage 2, both groups continued their respective administration and simultaneously added intranasal oxytocin spray. The treatment proceeded for a total of 28 weeks with 3 visits for outcomes measurement at 0, 16 weeks, and 28 weeks (V1, V2, and V3, respectively). While we originally planned to conduct the study outcome measurements at weeks 0, 12, and 24, we decided to prolong the study treatment for stage 1 for an additional 4 weeks based on some preliminary results. Such a change was justified by our determination that prolonged treatment of probiotic supplementation with the current strain of interest has not been previously investigated [5].

This study was conducted according to the guidelines described in the Declaration of Helsinki. Ethical approval of this study was issued by the Internal Review Board (IRB) of Massachusetts General Hospital (2017P001667). The clinical trial was registered through ClinicalTrials.gov (accessed on 2 May 2021) with identifier NCT03337035. Written informed consent was obtained from competent adult subjects or from the parents or legal guardians of children and adults with cognitive impairment according to the Internal Review Board (IRB) requirements. The protocol of this study was published previously [6].

Compliance and safety assessments of potential adverse effects were assessed monthly via telephone check-in and self-report via Internet Research Electronic Data Capture (REDCap, v9.5.23) software. All adverse events were reported to the Human Research Committee of Massachusetts General Hospital promptly in accordance with guidelines. The Data and Safety Monitoring Plan (DSMP) was in place and approved by IRB to ensure the safety of participants, the validity of data, and the appropriate termination of this study.

### 2.2. Participants

Study participants were recruited through advertising posters/flyers in local communities and through ASD parent networks and workshops. Participants were included if they were 3–25 years old and had a pre-existing diagnosis of ASD confirmed by the Diagnostic and Statistical Manual of Mental Disorder (DSM-IV TR/-5) criteria, Autism Diagnostic Observation Schedule, Second Edition (ADOS-2), and/or The Autism Diagnostic Interview-Revised (ADI-R). Other inclusion criteria are: participants must have stable medications for at least 4 weeks, have no planned changes in medications or psychosocial interventions during the trial period, are willing to provide stool samples and blood in the timely manner, and are willing to participate in interviews and study procedures. A potential participant was excluded if the subject was pregnant (before or during the study); had comorbidity of other neurological and/or psychiatric disorders, such as bipolar disorders or history of a substance use disorder; was on psychotropic medications; had an active cardiovascular disease that is not controlled by medication; or had received oxytocin or probiotic treatment within the last 4 weeks. The participants were interviewed and tested in the private room of the clinical research setting of Athinoula A. Martinos Center at Massachusetts General Hospital.

### 2.3. Randomization and Blinding

Randomization and allocation concealment were performed by a statistician who was not part of the research team, in collaboration with the Massachusetts General Hospital research pharmacy. Randomization sampling numbers were electronically generated, and central randomization at the research pharmacy using coded drug containers that are identical in appearance were prepared by the pharmacy to ensure allocation concealment. Blinding was maintained by making the capsules look identical. Both participants and the research staff who collected the outcome data were blinded to treatment status.

### 2.4. Interventions

*Lactobacillus plantarum* PS128 (PS128), which was isolated from a traditional Taiwan fermented mustard food [17], was deposited under DSMZ Accession No. DSM 28632. The genome architecture of PS128 was illustrated [18]. Both animal and human studies with PS128 demonstrated great safety [5,12,13,19]. The probiotic capsule contained only PS128 as a single-strain probiotic. Dosage in the study was 2 capsules a day (6 × 10^10^ CFUs). Microcrystalline cellulose capsules were used as a placebo for PS128. Both probiotics and placebo capsules were free gifts obtained from Bened Biomedical Co., Ltd (Taipei, Taiwan).

In this study, oxytocin was administered nasally. The Syntocinon^®^ Spray (Novartis Pharma AG; purchased from Apotheke Roter Ochsen, Schaffhausen, Switzerland and Victoria Apotheke Wholesale, Schwerzenbach, Switzerland) is currently the most commonly used standardized oxytocin nasal spray for clinical trials worldwide. We instructed the patient and family members about the use of this spray. Dosing began with 1 puff of 4 IU daily for the first week of the second stage. Subsequently, the dosage was increased to 1 puff per nostril daily (8 IU/d) for the second week and 1 puff per nostril twice a day (16 IU/d) for the third week. The dosage was then titrated up to the maximum dose of 32 IU daily, which is 2 puffs per nostril twice a day, starting on the fourth week. The dosage of 32 IU per day has been approved as safe and adequate in even younger patients (age 3–8 years) by a previous publication [20]. Another study reported a 4-week intranasal OXT treatment (24 IU, twice daily with total 48IU per day, which is more than the max dose in this study of 32 IU per day) in 32 children with ASD, aged 6–12 years old [21]. We achieved an active IND from the FDA, and the IND number is 138827 for Syntocinon^®^ (Pitocin, Oxytocin).

### 2.5. Outcomes

#### 2.5.1. Primary Outcome Measures

We evaluated two primary outcome measures:

1. Change in caregiver-rated Social Responsiveness Scale (SRS) [22];
2. Change in caregiver-rated Aberrant Behavior Checklist (ABC) [23].

The SRS is used to assess social interest and interaction based on five subscales. We interviewed all subjects older than 4 years. The ABC is an informant rating instrument that was empirically derived by a principal component analysis. It contains 58 items that resolve onto five subscales. We interviewed all of the subjects older than 5 years.

#### 2.5.2. Secondary Outcome Measures

- Blood sample collection and circulating biomarker analysis

Participants presented to the Athinoula A. Martinos Center for the visit after an 8 h fast three times (week 0, week 16, and week 28). Blood was drawn and processed to obtain serum, labelled with a unique code, and stored at −80 °C. Circulating serum OXT, myelin basic protein (MBP), glial fibrillary acidic protein (GFAP), S100 calcium-binding protein B (S100B), and interleukin-1β (IL-1β) were measured by ELISA (R&D Systems Inc., Minneapolis, MN, USA), following the manufacturer’s instructions.

- GI symptom severity assessments

GI symptoms were assessed by the validated GI severity index (GSI), including constipation, diarrhea, stool consistency, stool smell, flatulence, abdominal pain, unexpected daytime irritability, night-time awakening, and abdominal tenderness. The stool status was scored using the Bristol Stool Chart.

- Clinical Global Impression (CGI)

The clinical global impression (CGI) scale was developed for use in clinical trials to provide a brief, stand-alone assessment of the clinician’s view of the patient’s global functioning changes with the study medication. The CGI comprises two companion one-item measures evaluating the following: (a) severity of psychopathology from 1 to 7 (CGI-S) and (b) the improvement or change from the initiation of treatment on a similar seven-point scale (CGI-I) [24].

### 2.6. Stool Sample Processing

Stool samples were collected with an OMNIgene Gut OMR-200 collection kit (DNA Genotek Inc.) by the participants at home under the supervision of trained parents and stored at room temperature, before de-identification and delivery or shipment to the Athinoula A. Martinos Center, where stool samples were stored at −80 °C freezer. After all of the experiment samples were collected after week 28, they were hand delivered with dry ice packaging to a laboratory at Brigham & Woman’s Hospital for DNA extraction and sequencing analysis.

Microbial DNA was then extracted according to the manufacturer’s instructions, and DNA samples were quantified with a NanoDrop spectrophotometer. A260/A280 ratios were also measured to confirm high-purity DNA yield. Microbial 16S rRNA V4 genomic regions from total gut DNA samples were amplified with the following primers: 515F (AATGATACGGCGACCACCGAGATCTACACNNNNNNNNTATGGTAATTGTGTGCCAGCMGCCGCGGTAA) and 806R (CAAGCAGAAGACGGCATACGAGATNNNNNNNNAGTCAGTCAGCCGGACTACHVGGGTWTCTAAT). PCR products were purified and analyzed using a Bioanalyzer DNA kit, followed by quantification with real-time PCR. DNA libraries were pooled and sequenced on an Illumina MiSeq next-generation sequencing system (Illumina; San Diego, CA, USA) using a V4 2 × 250 bp paired-end protocol with overlapping reads.

### 2.7. Statistical Analysis

Data analyses were performed based on the intention-to-treat principle. The primary outcomes for the treatment comparisons were the changes in the scores of SRS and ABC (SRS T-score, ABC T-score). The secondary outcomes measurement includes CGI, GSI, levels of serum markers, and the gut microbiome.

An independent sample *t*-test/Wilcoxon rank-sum test for continuous variables was used to detect between-group differences in the measurement changes over the intervention course. Paired sample *t*-test and Wilcoxon signed-rank test were used to test the within-group difference in the primary outcomes and secondary outcomes before and after the intervention (V2-V1 and V3-V1). The z-test for equality of proportions without continuity correction was applied to differences in the proportion of subjects displaying change in secondary outcome measures.

We additionally performed a stratified analysis based on baseline SRS/ABC score, GI condition, and neuroinflammation/neuro-injury serum marker levels.

Sequencing data were processed and analyzed with a QIIME 2 [25], and α-diversity was calculated by Chao-1, Faith PD, Evenness, and observed OTUs using the Phyloseq R package. β-diversity, weighted UniFrac, unweighted UniFrac, Bray–Curtis, and Jaccard were analyzed.

SparCC (Sparse Correlations for Compositional data) co-abundance networks were constructed to examine the longitudinal change in associations between gut microbiota [26]. Correlations with magnitudes greater than a SparCC cutoff of 0.7 were considered significant. Identified hub taxa and the respective hub scores are indicated by the size of the circle. False discovery rate (FDR)-based type 1 error control was made per study visit on a group-wise basis across all assessed variables. This was conducted via MaAsLin2 for assessed correlations between primary and secondary outcomes and blood serum marker concentrations against microbiota relative abundances [27]. Significant correlations were considered at an FDR < 0.1.

PICRUSt (phylogenetic investigation of communities by reconstruction of unobserved states) is a computational approach to predict the functional composition of a metagenome using marker gene data and a database of reference genomes and was applied to the current 16S dataset. The relative change in abundance of each feature abundance (ASVs or pathways) between visits V1 and V2 (V2-V1) and visits V1 and V3 (V3-V1) were computed for each experimental subject. Then, differential analyses were performed on the relative changes between the probiotics and placebo groups with Wilcoxon rank-sum test. 

## 3. Results

### 3.1. Demographics

**Figure 1:**
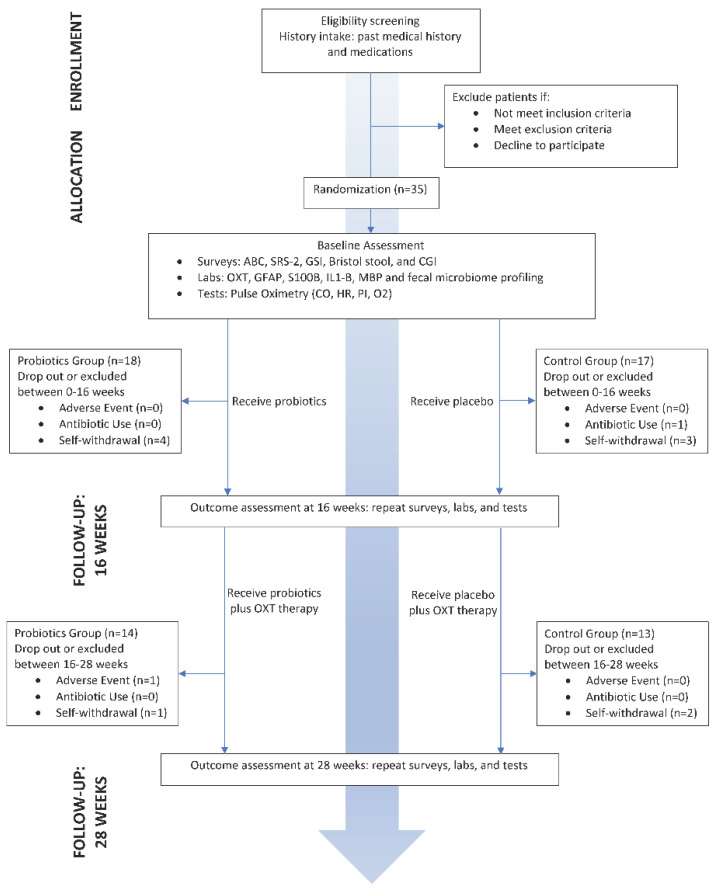
Flowchart of overall study design and conduct.

The flowchart of the study is shown in Figure 1. Between 12 December 2018 and June 17, 2019, we enrolled and randomized 35 patients with ASD aged 3–20 years (median = 10.30 years; 26 males, 9 females). The placebo group subjects had an age range of 4.69–19.70 years (10.70 ± 4.76 years; 11 males, 6 females), while the probiotic group subjects had an age range of 3.60–18.50 years (9.85 ± 4.91 years; 15 males, 3 females). The baseline demographic features and clinical indices of the 35 participants are summarized in Table 1. ASD severity measures via ABC, SRS, and CGI-S of all participants suggest that the baseline severity is determined to be 275 ± 32.3 via the ABC standardized score (T-score), 82.6 ± 11.6 via the SRS standardized score (T-score), and 5.11 ± 1.02 via the CGI-S. Group-wise comparisons of such scores are also summarized in Table 1. There were no significant differences between the two groups in these demographic and clinical indices (*p* > 0.05). No serious or severe adverse events were observed. One subject was terminated due to minor nose bleeding in stage 2 that resolved quickly on its own; this subject had a history of recurrent nose bleeding related to their seasonal rhinitis. Another subject was terminated due to oral ambulatory antibiotics use for a mild upper respiratory infection. Other self-withdrawals were due to moving, travel, or other administrative reasons which were found to have no relation to the study or any adverse events. There was no significant difference of dropouts found between the two groups (*p* > 0.05).

### 3.2. Socio-Behavioral Parameters and Other Clinical Indices

Changes in socio-behavioral parameters as measured by ABC and SRS from visit 1 to visit 2 (V2-V1) for the control group and probiotics group, and from visit 1 to visit 3 (V3-V1) for the OXT group and the probiotic + OXT combination group (Table 2). We performed independent Wilcoxon rank-sum tests for subjects in each treatment group against the control group subjects. Trends of improvement in the total ABC score (*p* = 0.077), stereotypic behavior score (*p* = 0.069), and SRS cognition score (*p* = 0.059) were observed in the combination therapy group (Probiotic + OXT), although no significant differences were observed in the total scores or subscales of the ABC and SRS (Wilcoxon rank-sum test, *p* > 0.05).

**Figure 2:**
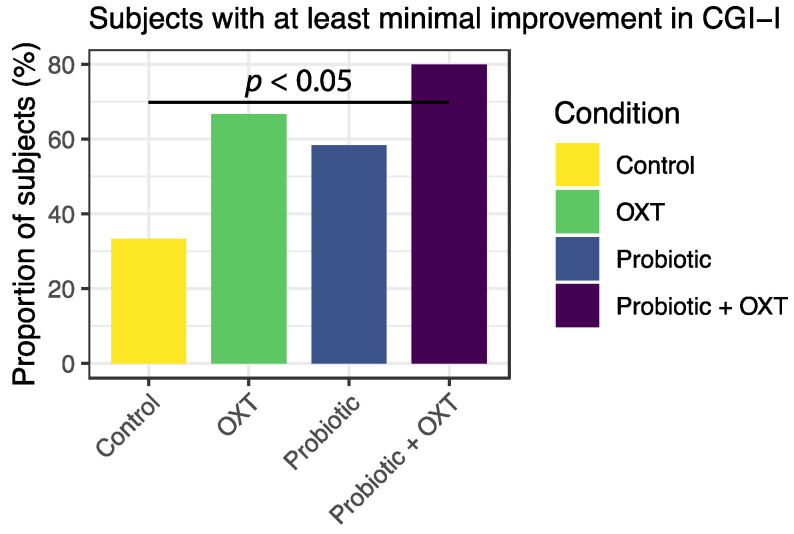
Proportion of subjects displaying improvement in CGI-I overtime among all subjects within a treatment condition. The z-test for equality of proportions is applied and the number of subjects displaying at least minimal improvement (CGI-I ≤ 3) in the control condition and the probiotic + OXT condition is significantly different (*p* < 0.05), while the changes of probiotic and OXT alone groups are non-significant, though a trend of improvement is seen in both intervention groups.

CGI was assessed to evaluate ASD symptoms and the relative extent of improvement in symptoms at each visiting time. As seen in Figure 2, the proportion of subjects showing improvement is significantly increased only in the probiotic + OXT combination group when compared against that of the control group (Pearson’s χ^2^-test, *p* < 0.05), while the changes in the probiotics or OXT alone groups were non-significant, though a trend of improvement was observed in both intervention groups. The waterfall data of the CGI score reduction in each group is shown in supplement Supp. Figure S1.

GIS showed no significant changes in the three treatment groups compared with the placebo group over the treatment course (*p* > 0.05).

### 3.3. Gut Microbiome

**Figure 3:**
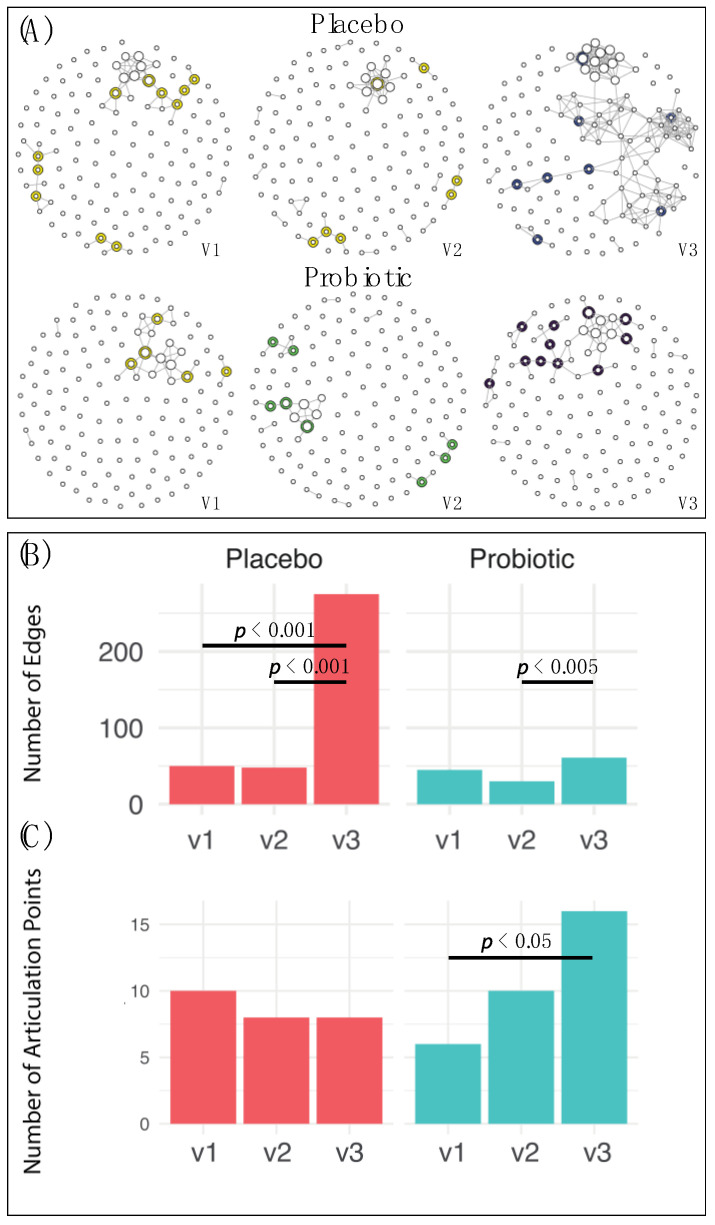
SparCC network associations between genus-level gut microbiota between subjects receiving placebo and those receiving the active probiotic overtime, using a SparCC cutoff of 0.7. Placebo group V1 is baseline, V2 is after placebo, V3 is after placebo added OXT; probiotics group V1 is baseline, V2 is after probiotics, V3 is after probiotics added OXT. (**A**) SparCC co-occurrence network. Articulation points are marked as halos around the node. Hub score is indicated by the size of the node. (**B**) The number of lines or edges is significantly enriched in both the OXT alone and combination groups at visit 3 (Pearson’s χ^2^-test with Yates’ continuity correction, *p* < 0.005). (**C**) The number of articulation points is only significantly increased in the combination group at V3 compared to baseline number of articulation points (Pearson’s χ^2^-test with Yates’ continuity correction, *p* < 0.05).

The gut microbiome was investigated by sequencing the fecal DNA. Although α- and β-diversity showed no significant changes in this study (Supp. Figure S2), we found a significant increase in microbiota hubs and numbers of connection edges uniquely at V3 as compared to the two previous visits V1 and V2 (Figure 3A), using a SparCC cutoff of 0.7. The lines or edges of the connections were significantly increased in both the OXT alone group (*p* < 0.001) and the combination group (*p* < 0.005, Figure 3B), however, the number of articulation points (those with halos around the node also called “hubs”) were only significantly more in the combination group (Pearson’s χ^2^-test with Yates continuity correction, *p* < 0.05, Figure 3C).

When we investigated those key hub taxa with a hub score greater than 0.8, interestingly, we found a distinct panel of hubs (marked as “+”) in the three treatment groups without overlaps. *Christensenellaceae R7*, *Ruminococcaceae UCG-002*, *Lachnospiraceae UCG-001*, *Blautia*, and *Barnesiella* were only present in the combination therapy group; distinct hubs, *Coprococcus-2*, *Rikenellaceae RC9*, *Bilophila*, *Catenibacterium*, and *Holdemanella*, were only found in the OXT alone group; while *Roseburia*, *Veillonella*, and *Streptococcus* were only present in the probiotics group. None of the key hubs were only found in the placebo group (Supp. Table S1).

**Figure 4:**
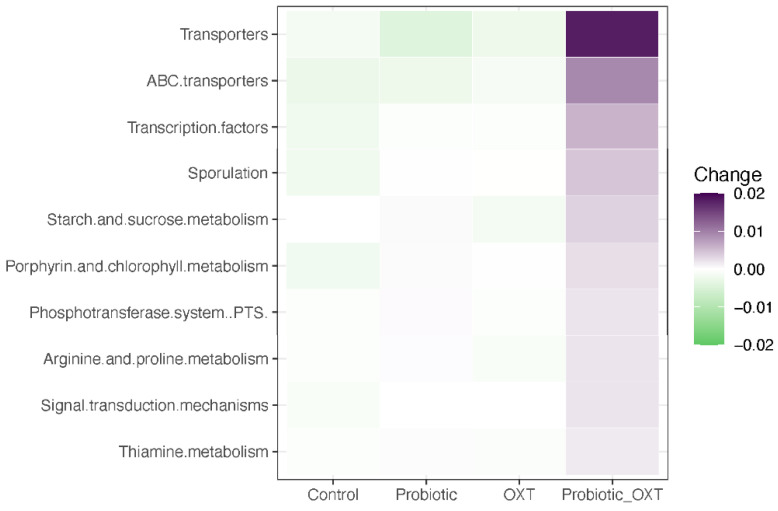
Heatmap of mean change in predicted functional profile based on gut microbiota abundance across four study groups. Shown changes in functional profiling indices demonstrated more changes in the combination group, although these changes are not significantly different when compared to the control group (*p* > 0.05).

Functional gene predictive analysis indicated that several genes trended towards greater abundances in the combination group over the 28-week treatment period. Notably, genes encoding transporters, ABC transporters, transcription factors, sporulation, starch and sucrose metabolism, porphyrin and chlorophyll metabolism, signal transcription metabolism, arginine and proline metabolism, and thiamine metabolism were found to be more enriched in combination groups than the other groups, although the difference was not statistically significant (*p* > 0.05, Figure 4).

We then performed the Spearman correlation analysis to assess the correlation between socio-behavioral parameters measured by the ABC and SRS and microbiota relative abundance at baseline and over the course of treatment. Interestingly, the taxa *Eubacterium hallii* group was found to be significantly associated with total scores (R = −0.59, false discovery rate-adjusted *P* (FDR) = 0.00767) and three subscales of the SRS before treatment (SRS communication: R = −0.55, FDR = 0.04282); SRS mannerism: R = −0.6, FDR = 0.01753; SRS motivation: R = −0.56, FDR = 0.0645; Table 3); the strongest negative correlation was found between the *Eubacterium hallii* group and the SRS cognition score (Spearman’s rho = −0.97, *p* = 0.0048, FDR < 0.1). Furthermore, the absolute change (V3-V1) in *Eubacterium hallii* group abundance in the combination therapy group is positively correlated with the baseline SRS cognition score (Spearman’s rho = 0.71, *p* = 0.05), meanwhile, the absolute change (V3-V1) in *Rikenelaceae*, *Alistipes*, *Christensenellaceae R7*, and *Ruminococcaceae UCG-002* in the combination therapy group positively correlated with the ABC stereotypic behavior score at baseline (Table 4). Of note, *Rikenelaceae* and *Alistipes* were found to be significantly correlated with SRS motivation at baseline (Table 3), while *Christensenellaceae R7* and *Ruminococcaceae UCG-002* are two out of five important and unique hubs found only in the combination treatment group (Supp. Table S1). Additionally, *Lachnospiraceae (uncultured)* was found to be negatively correlated with the ABC inappropriate speech at baseline (R = −0.68, FDR = 0.04247).

### 3.4. Blood Serum Markers

**Figure 5:**
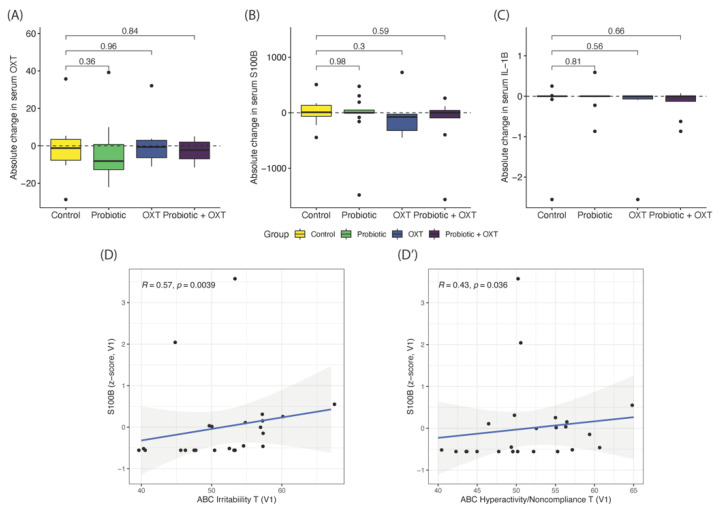
Summary of longitudinal serum marker changes and associated correlations. Absolute changes in serum OXT (**A**), S100B (**B**), and IL-1β (**C**) levels, comparing each treatment group against controls. Baseline serum S100B is positively correlated with ABC irritability T (**D**) and ABC hyperactivity/noncompliance T-scores (**D’**).

For the OXT level as measured, there were no significant changes of the four groups (*p* > 0.05) (Figure 5A). For the inflammatory markers tested in this study, a trend of greater decrease in S100 in the OXT alone group (Figure 5B) and IL-1β levels in the combination therapy group (Figure 5C) were observed; however, these differences were not statistically significant (Wilcoxon rank-sum test, *p* > 0.05). By the Spearman correlation analysis, we also found that the S100 level positively correlated with ABC irritability (Figure 5D) and ABC hyperactivity/non-compliance scores (Figure 5D) at baseline.

## 4. Discussion

In this pilot study, we explored and compared two promising interventions, probiotics and oxytocin, both alone and in combination, against placebo controls. All interventions were well tolerated, and no major adverse events were observed. Only in the combination treatment group, we observed a trend of improvement in social and behavioral measurements (ABC and SRS), particularly in the ABC total score (*p* = 0.077), ABC stereotyped behavior sub-score (*p* = 0.069), and SRS cognition sub-score (*p* = 0.059). Meanwhile, a significant improvement of CGI was found only in the combination treatment group compared to the placebo, not in the probiotics or the OXT treatment alone groups. CGI provides a brief, stand-alone assessment of the clinician’s view of the patient’s global functioning prior to and after initiating a study medication. The CGI-I represents the change from the initiation of treatment on a seven-point scale [24]. In this study, the CGI-I was conducted by the clinician, who was totally blinded in treatment status and was also well acquainted with the subjects. Our finding that combination therapy elicited significant clinical improvement has not been reported previously. Previously, PS128 was found to increase dopamine and serotonin in different animal studies [12,13]; however, its relationship with oxytocin has not been tested. A potential mechanism of induction for increased serotonin secretion posits that bacterial tryptophan secretion catabolites may interact with intestinal enteroendocrine cells, thereby increasing intestinal motility and modulating the central nervous system (CNS) [28]. Furthermore, there is growing evidence for crucial interactions among the dopaminergic system, oxytocin/vasopressin, and serotoninergic systems in different areas of the brain that greatly influence human social behavior [29,30]. We believe that this finding not only opens a new avenue for ASD treatment but also furthers our knowledge about the gut–brain axis and ASD pathogenesis and warrants further studies.

Building on our finding of psychopathology improvement with combination therapy, we found some significant favorable changes in the gut microbiome over the intervention course. In particular, a significant increase in the SparCC co-occurrence network was found. The lines of the connections were significantly increased in both the OXT alone group (*p* < 0.001) and the combination group (*p* < 0.005), however, the number of articulation points (hubs) were only significantly more in the combination group (*p* < 0.05) not in the OXT alone group (*p* > 0.05), which suggests more critical and meaningful microbiome interactions are involved in the combination therapy. It is well known that an articulation point in a network is a node whose removal disconnects the network. This new finding favors the synergistic effects of the combination therapy. When examining the driving species of articulation points, we observed that the identified microbiota in the combination treatment group is unique not only from placebo group but also without overlaps with either the probiotics or the OXT alone group. Among those with a high hub score (>0.8) in the combination therapy group, both *Blautia* and *Barnesiella* were previously reported to be reduced in the gut of ASD patients [31,32,33], and both can promote butyrate production, which benefits gut health [34]. *Christensenellaceae* R7, *Ruminococcaceae* UCG-002, and *Lachnospiraceae* UCG-001 have not yet been reported in ASD, but their health benefits related to weight, gut health, and diabetes have been reported. The enrichment of these hubs overall favors improving metabolism and inflammation [35,36,37,38,39]. The findings obtained from the OXT alone group with high score hubs of *Bilophila*, *Coprococcus-2*, *Holdemanella*, Rikenellaceae, and *Catenibacterium* also favor anti-inflammation and gut health in general [5,40,41,42,43,44,45]. Similarly, *Roseburia, Veillonella*, and *Streptococcus*, which were found to have high hub scores in the probiotics group, also promote anti-inflammation, gut health, and additionally, carbohydrate metabolism [5,40,46,47]. In the combination group, significantly increased numbers of the articulation points are likely contributing to their better treatment responses than each treatment alone; the distinct hub panel from the single therapy groups also supports the synergistic effects as observed in the combination therapy group. The advance of network theory helps to disentangle the higher order interactions that occur within microbiomes [48], which could be more important than the microbiome diversity representation.

Additionally, predicted functional gene analysis suggested that several important pathways are more activated in the combination group than the placebo and other treatment groups. The pathways with the highest discriminative power in the combination group were “Transporters” followed by “ABC transporters”, transcription factors, starch and sucrose metabolism, arginine and proline metabolism, and thiamine metabolism. ABC transporters couple energy metabolism and mediate the uptake of nutrients and physiological functions, which were found to be repressed in an ASD model with impairment of the neuronal network [49,50]. The starch and sucrose metabolism pathways have been found to be down-regulated in ASD [51]. The human gut microbiome is a critical component of digestion, as it facilitates the breakdown of complex carbohydrates and proteins [52]. Arginine has been shown to be substantially reduced in cases of gut inflammation and infection [53]. As a metabolic precursor for nitric oxide (NO), it regulates neuron survival, differentiation, synaptic activity, and plasticity [54]. Thiamine (vitamin B_1_) is an essential cofactor that when deficient, contributes to symptoms such as confusion, reduced memory, and sleep disturbances [55], and when adequately concentrated, promotes homeostasis of a healthy gut ecosystem. These favorable findings further supported the use of combination therapy as a promising treatment approach than using them alone, as a synergistic effect was involved to facilitate energy metabolism and normal physiological functions.

Importantly, gut microbiome was found to be highly correlated with social behavioral parameters. *Eubacterium hallii* was found to be significantly negatively correlated with the SRS total score and sub-scores, particularly the SRS cognition sub-score. More enriched gut *Eubacterium hallii* abundances correlate with lower SRS scores, thus representing a better social function level. This strong correlation is not only observed at baseline but also with the absolute increase in the combination group at visit three. As mentioned earlier, the improvement of the SRS cognition subscale in the combination group, as shown in Table 2 (*p* = 0.059), is one of the most prominent trends of improvement observed. The higher the *Eubacterium hallii* at baseline, the more favorable the improvement of social cognition over the course of the combination treatment. The lower the level of social cognition (with a higher score) at baseline, the more an increase in *Eubacterium hallii* in the combination group was observed. *Eubacterium hallii* can utilize glucose, and the fermentation intermediates acetate and lactate to form butyrate, which benefits gut health [56,57]; however, this promising taxon has not yet been reported in ASD patients. Additionally, *Christensenellaceae* R7 and *Ruminococcaceae* UCG-002, two of the five unique hubs that were only observed in the combination treatment group (V3-V1), were found to be positively correlated with the ABC stereotypic behavior sub-score, which describes one of the ASD core symptoms. This correlation analysis further demonstrates the strong association of gut microbiome with ASD core symptoms at baseline and after a favorable treatment response in the combination group.

To further our knowledge of these treatment responses, we also measured serum oxytocin and inflammatory markers over the course of the treatment. In this study, we did not find significant changes of oxytocin level in the three treatment groups when compared with the placebo group.

The aberrant OXT serum levels have been reported in ASD individuals to varying degrees, sometimes decreased [58,59], sometimes no difference [60,61], and sometimes enriched compared to non-ASD controls [62]. These differences could be related to subsets of the ASD population with reduced biosynthesis or release of OXT [63,64], dysfunctional OXT processing dysfunction, or oxytocin receptor abnormalities [65]. Further studies are warranted to investigate these potential ASD subtypes and to resolve these variable results and treatment responses in different subsets.

Inflammatory mechanisms linked with ASD have been widely reported. Inflammatory cytokines were found to be significantly elevated in ASD individuals compared with healthy controls [66,67]. Similarly, brain injury and inflammatory markers, GFAP, MBP, and S100B, have been found to be significantly enriched in ASD children than controls [67,68,69,70,71]; these brain injury markers and cytokine release subsequently trigger glial cell activation and the inflammatory process in the brain [72]. In this study, we tested these four serum inflammatory markers and found that a trend of decrease in S100 in the OXT group, and the decrease in IL-1β to be more pronounced in combination treatment. S100B was shown to have a significant positive correlation with the severity of problem behaviors (ABC irritability and hyperactivity scores at baseline; *p* < 0.05).

There are several limitations of the study that deserve consideration. (1) Despite our adoption of proper recruitment and retention strategies, the participant enrollment and retention for this trial were challenging. A relatively small sample size in this pilot trial limited the statistical power and further subgroup analysis. (2) Although there was no statistical difference in clinical indices between the probiotics and placebo groups at baseline, the wide age range used in this study resulted in high subject population heterogeneity and potentially variable treatment efficacy. Future studies with a larger sample size and subgroup stratification are warranted. (3) Due to considerable Asian and other minority patients with some cultural and language barriers, in addition to multiple influencing factors on behavioral variabilities, the parent rating of social behavioral scales may be somewhat biased. (4) Sequential comparisons were not made at the same time point for the four intervention groups. The two-stage design seems inferior to simply having four groups with a 2 × 2 factorial design; in this design, the prolonged treatment course might be influenced by other randomly occurring factors.

## 5. Conclusions

In the present pilot trial, we demonstrate that the concurrent supplementation of oral probiotic *Lactobacillus plantarum* PS128 and intranasal OXT in participants with ASD may reduce ASD core socio-behavioral symptoms and clinical global functioning. Statistically significant improvements in ASD-related outcomes over the treatment course via combined therapy are attributed to the proposed synergistic interactions between the two treatments, which are mediated via the gut–brain axis. Furthermore, participants receiving combined therapy showed significant improvements in gut microbiome dysbiosis characterized by several distinct hub networks. Despite such promising preliminary findings, the underlying mechanisms and causal relationships of such synergistic effects remain elusive and deserve further investigation in large-scale and well-designed trials.

## Figures and Tables

**Table 1:** Summary of subject demographics and clinical indices at baseline.

	Placebo (*n* = 17)	Probiotic (*n* = 18)	*p*-Value *
**Demographic**			
Age (Mean ± SD)	10.7 ± 4.76	9.85 ± 4.91	0.66
Sex (*N*, %)			
Male	11 (64.7%)	15 (83.3%)	0.38
Female	6 (35.3%)	3 (16.7%)	
Ethnicity (*N*, %)			
Asian	14 (82.4%)	14 (77.8%)	0.62
Hispanic	0	1 (5.5%)	
White	3 (17.6%)	3 (16.7%)	
**Clinical Indices**			
GI Severity Index (Mean ± SD)	3.33 ± 1.37	2.54 ± 2.03	0.18
Stool Type (Bristol stool chart; *N*, %)			
Type 1&2 (Constipated)	1 (5.9%)	1 (5.6%)	1.00
Type 3&4 (Normal)	12 (70.6%)	13 (72.2%)	
Type 5, 6, 7 (Loose Stool)	3 (17.6%)	3 (16.7%)	
ABC Standardized Score (T-score, Mean ± SD)	278 ± 34.8	272 ± 30.2	0.38
SRS Standardized Score (T-score, Mean ± SD)	83.0 ± 12.1	82.3 ± 11.5	0.96
CGI-S (Mean ± SD)	5.12 ± 1.17	5.11 ± 0.90	0.97

* Continuous data was evaluated for *p*-values via the Wilcoxon rank-sum test while categorical data was evaluated for intergroup differences via the Pearson’s χ^2^-test with Yates’ continuity correction.

**Table 2:** Summary of improvement in socio-behavioral measures. Data are presented as mean change ± SD.

	Improvement in Score (Mean Change ± SD)				*p*-Value *		
	Control	Probiotic	OXT	Probiotic + OXT	Probiotic	OXT	Probiotic + OXT
**ABC**							
Total Score	15.00 ± 26.75	6.67 ± 26.00	12.33 ± 23.16	−10.43 ± 31.91	0.48	0.84	0.077
Irritability (S1)	3.45 ± 6.67	−0.92 ± 6.20	2.17 ± 3.97	−2.43 ± 9.86	0.19	0.84	0.20
Social Withdrawal (S2)	1.82 ± 8.30	3.50 ± 6.36	1.67 ± 7.92	−4.00 ± 10.26	0.46	1	0.28
Stereotypic Behavior (S3)	3.18 ± 4.38	1.17 ± 6.64	1.83 ± 5.78	−1.29 ± 4.27	0.67	0.45	0.069
Hyperactivity/Noncompliance (S4)	5.64 ± 8.31	2.33 ± 8.91	5.50 ± 7.45	−1.57 ± 10.50	0.34	0.84	0.16
Inappropriate Speech (S5)	0.91 ± 2.12	0.58 ± 1.78	1.67 ± 3.31	−1.14 ± 3.02	0.66	0.80	0.20
**SRS**							
Total Score	22.09 ± 23.71	12.31 ± 22.21	10.00 ± 24.71	4.88 ± 22.95	0.45	0.23	0.26
Awareness	1.18 ± 2.36	1.15 ± 2.58	1.33 ± 2.58	0.13 ± 3.04	0.86	0.96	0.28
Cognition	4.73 ± 4.54	0.92 ± 4.31	2.00 ± 5.18	0.38 ± 5.53	0.15	0.15	0.059
Communication	7.09 ± 9.45	4.46 ± 7.13	0.50 ± 9.09	2.00 ± 8.11	0.68	0.11	0.36
Motivation	3.27 ± 3.58	2.54 ± 4.96	2.83 ± 5.67	0.88 ± 6.15	0.77	0.61	0.16
Mannerisms	5.82 ± 6.82	3.23 ± 6.61	3.33 ± 6.09	1.50 ± 5.50	0.58	0.39	0.20

* Provided *p*-values are based on Wilcoxon rank-sum test between the mean improvement in score in the control group and the respective treatment groups.

**Table 3:** Spearman correlations between the microbiota relative abundance and socio-behavioral parameters before treatment for all subjects.

Clinical Feature	Microbiome Taxa	*R*	FDR *
ABC Inappropriate Speech (T)	*Lachnospiraceae* (uncultured)	−0.68	0.04247
SRS Communication (T)	*Eubacterium hallii Group*	−0.55	0.04282
SRS Mannerisms (T)	*Eubacterium hallii Group*	−0.60	0.01753
SRS Motivation (T)	*Rikenellaceae*	−0.58	0.0645
	*Alistipes*	−0.58	0.0645
	*Eubacterium hallii Group*	−0.56	0.0645
SRS Total Score (T)	*Eubacterium hallii Group*	−0.59	0.00767

* All presented correlations are significant at FDR < 0.1.

**Table 4:** Significant correlations between primary outcomes and microbiota relative abundance in probiotic group and placebo group subjects based on Spearman’s rank correlation.

Clinical Feature	Microbiome Taxa	Probiotic Group (*n* = 18)		Placebo Group (*n* = 17)	
		*R*	*p*-Value	*R*	*p*-Value
SRS Cognition T (V1)	*Eubacterium hallii* Group (V3-V1)	0.71	0.050	0.01	0.790
SRS Cognition T (V3-V1)	*Eubacterium hallii* Group (V1)	−0.97	0.005 *	0.17	0.750
ABC Stereotypic Behavior T (V1)	Rikenellaceae (V3-V1)	0.94	0.017	0.20	0.590
	*Alistipes* (V3-V1)	0.94	0.017	−0.02	0.960
	Christensenellaceae R-7 Group (V3-V1)	0.82	0.046	−0.09	0.810
	Ruminococcaceae UCG-002 (V3-V1)	0.83	0.058	−0.37	0.290

* FDR < 0.1 based on screening of results.

## Data Availability

The data presented in this study are available on request from the corresponding author.

## Supplementary Materials

The following are available online at https://www.mdpi.com/article/10.3390/nu13051552/s1, Figure S1: Waterfall plot of reduction in CGI-S score for each subject in different intervention groups, Figure S2: Overview of gut microbiome species diversity, Table S1: Summary of identified key hub taxa based on SparCC network analysis.
