# Supplementary material for: Probiotic and Oxytocin Combination Therapy in Patients with Autism Spectrum Disorder: A Randomized, Double-Blinded, Placebo-Controlled Pilot Trial
Source: Nutrients. 2021 May 5;13(5):1552. doi: 10.3390/nu13051552 (PMC8147925; doi:10.3390/nu13051552)
Supplement: Supplementary file 1 [file nutrients-13-01552-s001.zip › supplementary/Figure_S1_leg.pdf]

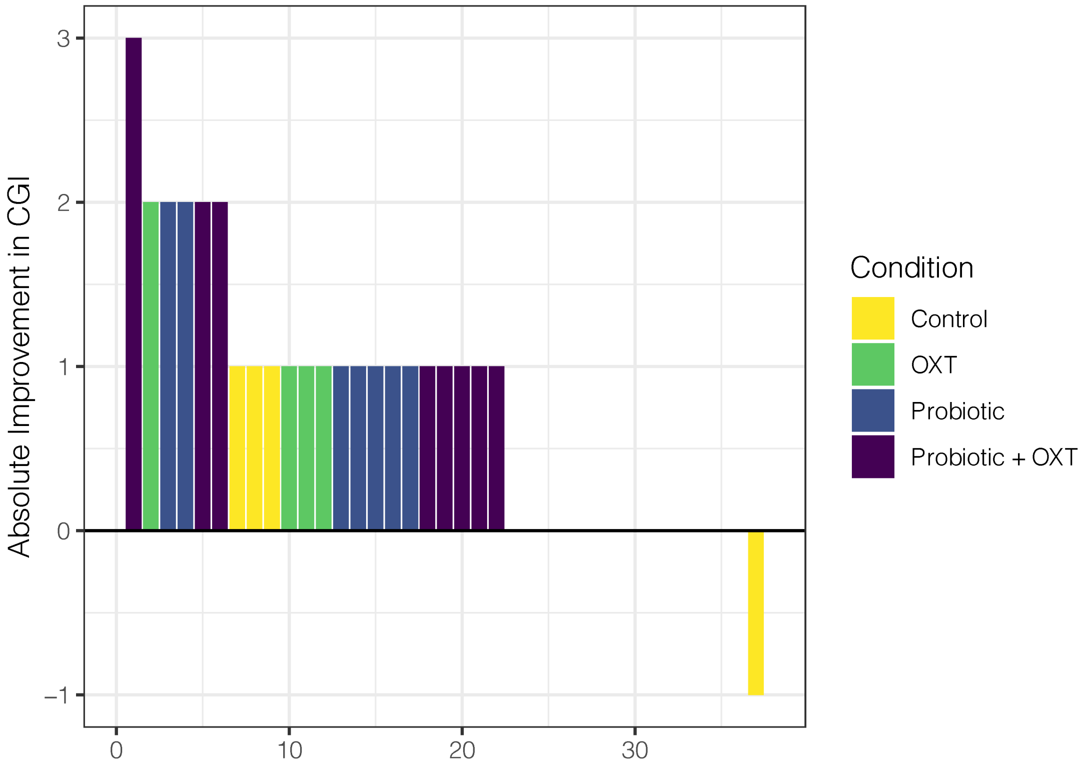

Supplementary Figure 1. Waterfall plot of reduction in CGI-S score for each subject in different intervention groups. Absolute improvement in CGI refers to the normalized scores of CGI-I (i.e., normalized CGI-I = -(CGI-I - 4).
