# Supplementary material for: Probiotic and Oxytocin Combination Therapy in Patients with Autism Spectrum Disorder: A Randomized, Double-Blinded, Placebo-Controlled Pilot Trial
Source: Nutrients. 2021 May 5;13(5):1552. doi: 10.3390/nu13051552 (PMC8147925; doi:10.3390/nu13051552)
Supplement: Supplementary file 1 [file nutrients-13-01552-s001.zip › supplementary/Figure_S2_leg.pdf]

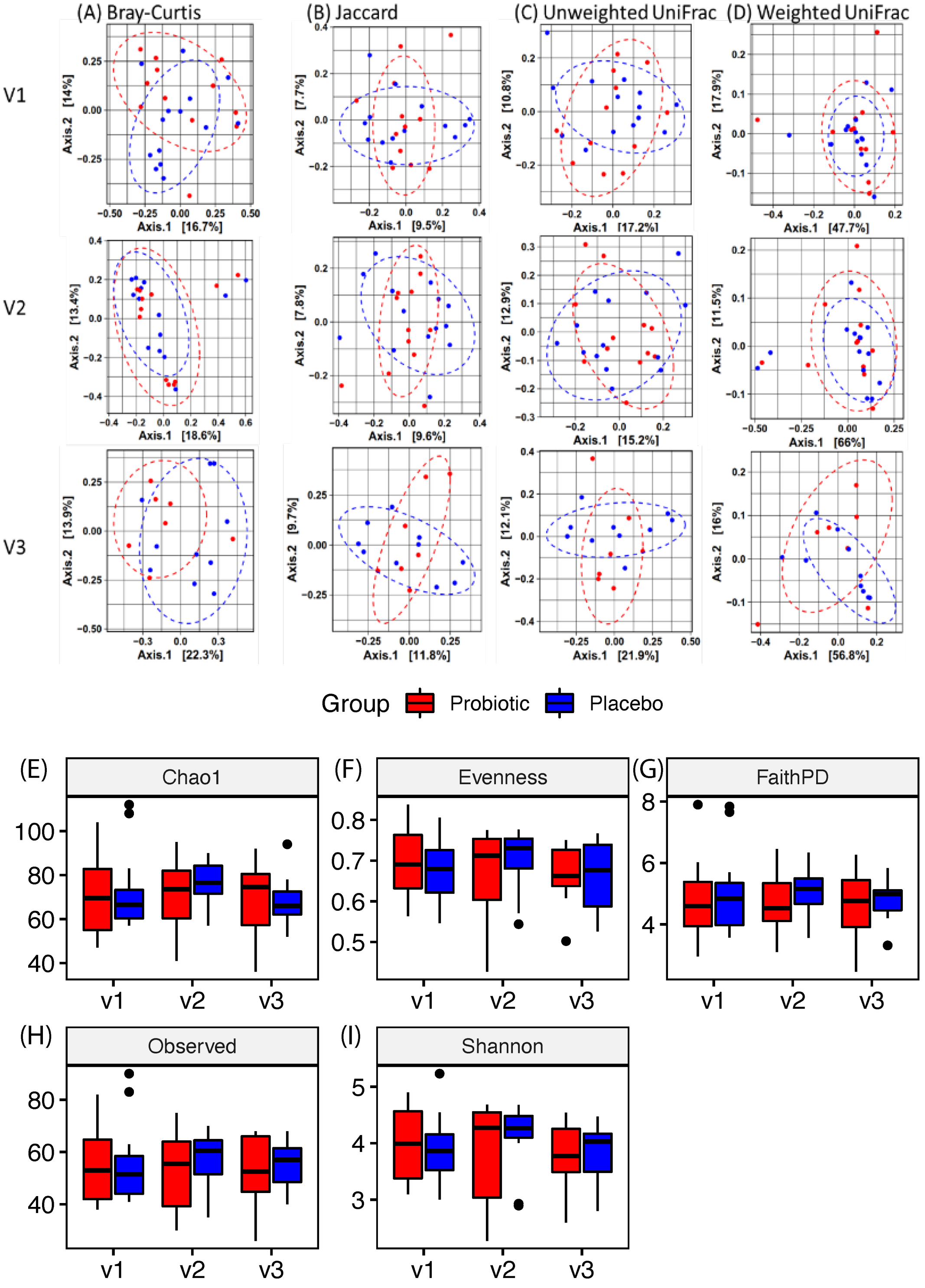

Supplementary Figure 2. Overview of gut microbiome species diversity. (A-D) PCoA of gut microbiome β-diversity metrics over the treatment course. (E-I) boxplots of α-diversity metrics per study visit.
