# Supplementary material for: Probiotic and Oxytocin Combination Therapy in Patients with Autism Spectrum Disorder: A Randomized, Double-Blinded, Placebo-Controlled Pilot Trial
Source: Nutrients. 2021 May 5;13(5):1552. doi: 10.3390/nu13051552 (PMC8147925; doi:10.3390/nu13051552)
Supplement: Supplementary file 1 [file nutrients-13-01552-s001.zip › supplementary/Table_S1.pdf]

Table S1. Summary of identified key hub taxa based on SparCC network analysis.

Taxa	Probiotic (Hub Score)			Placebo (Hub Score)		
	V1	V2 (Probiotic)	V3 (Probiotic + OXT)	V1	V2	V3 (OXT)
Roseburia	-	+ (0.9)	-	-	-	-
Streptococcus	-	+ (0.9)	-	-	-	-
Veillonella	-	+ (0.8)	-	-	-	-
Coprococcus 2	-	-	-	-	-	+ (1)
Rikenellaceae RC9 gut group	-	-	-	-	-	+ (1)
Bilophila	-	-	-	-	-	+ (0.9)
Catenibacterium	-	-	-	-	-	+ (0.9)
Holdemanella	-	-	-	-	-	+ (0.8)
Blautia	-	-	+ (0.8)	-	-	-
Barnesiella	-	-	+ (0.8)	-	-	-
Christensenel-laceae R7	-	-	+ (1)	-	-	-
Lachnospiraceae UCG-001	-	-	+ (0.8)	-	-	-
Ruminococcaceae UCG-002	-	-	+ (1)	-	-	-

"+" marks that a taxon of interest (hub score>0.8) is found within the top 10 hub taxa at the given study visit and experimental group; "-" marks not found. The provided value is the hub score (the cut off score is 0.8).
